# Structural considerations and differences between leaf canals and secretory cavities in Asteraceae

**DOI:** 10.1007/s00709-024-02028-8

**Published:** 2025-01-14

**Authors:** Daniel M. Martínez-Quezada, Alicia Rojas-Leal, José Luis Villaseñor, Teresa Terrazas

**Affiliations:** 1 Área de Biología, Departamento de Preparatoria Agrícola, Universidad Autónoma Chapingo, Carretera México-Texcoco Km 38.8, 56230 Texcoco, Mexico State Mexico; 2 Departamento de Botánica, Instituto de Biología, Universidad Nacional Autónoma de México Apartado Postal, 70-233, 04510 Mexico City, Mexico

**Keywords:** Secretory structures, Epithelial cells, Schizogenous, Lysigenous, *Bidens*, *Tagetes*

## Abstract

Secretory canals are distributed among seed plants, and their diversity is concentrated in many families of angiosperms, while other internal secretory structures such as secretory cavities have been identified only in Rutaceae, Myrtaceae, and Asteraceae. Identifying and recognizing these two types of secretory structures has been complicated, mainly due to their structural similarities. In this study, the ontogeny of canals and secretory cavities in two species of Asteraceae are described and compared, to understand the structural differences between them and allow the establishment of more appropriate homology hypotheses. Leaves of *Bidens odorata* and *Tagetes tenuifolia* in different stages of development, including the apex of the stems, were collected. The samples were processed using the methacrylate technique, and longitudinal and transverse sections were made. The development of both, canals and secretory cavities, is schizogenous, in contrast to what was previously reported for other families such as Rutaceae, where they are reported as lysigenous. In Asteraceae, canals originate from cells of the procambium while cavities originate from cells of the ground meristem. The structural and developmental similarities between both types of secretory structures allow us to infer that they have a close evolutionary origin. Canals and secretory cavities in Asteraceae can be differentiated based on the number of strata of secretory epithelium and sheath, the modifications of epidermal cells and mesophyll, and the type of promeristem that gives rise to them. Probably extravascular canals give rise to cavities in leaves of Asteraceae and probably in other plant families.

## Introduction

Secretory structures in vascular plants can be classified into two categories, external or internal (Dickison 2000); within the latter, there can be found canals, secretory cavities, and laticifers (Evert 2006). Traditionally, internal secretory structures were recognized by their secretions or structural characteristics, including the presence of specialized secretory cells called epithelial cells. In this sense, laticifers are constituted by elongated cells that can fuse forming simple or highly branched structures that produce generally whitish exudates when the organs break (Mauseth 1988). In contrast, secretory cavities and canals are structurally more complex as they are intercellular spaces surrounded by secretory epithelium and non-secretory cells forming a sheath (Martínez-Quezada et al. 2023). Canals have been reported in a large number of angiosperm families (Metcalfe and Chalk 1950; Fahn 1979), while secretory cavities have been typically identified in Rutaceae, Myrtaceae, and recently in Asteraceae (Kalachanis and Psaras 2005; Martínez-Quezada et al. 2023).

According to Esau (1977) and Fahn (1982), the main difference between canals and secretory cavities is their ontogenetic origin, schizogenous in the case of canals, and lysigenous for secretory cavities. However, there are some cases where this type of internal secretory structures shares developmental stages that are typically recognized in schizogenous or lysigenous patterns, which is why they are called schizolysigenous secretory structures (Turner et al. 1998; Prado and Demarco 2018). Ontogenetic studies have provided more information regarding the differences between canals and cavities, so according to these works, they can be differentiated based on their developmental patterns (Bezerra et al. 2018).

Canals and cavities are present in many angiosperm families, particularly those with great species diversity (Metcalfe and Chalk 1950). Canals have been reported in species belonging to 17 of the 40 tribes recognized in Asteraceae, while secretory cavities have been recorded only in members of the subtribe Pectidinae of the tribe Tageteae (Martínez-Quezada et al. 2023). These secretory structures are commonly confused both in Asteraceae and in other groups of angiosperms (Kalachanis and Psaras 2005; García-Sánchez et al. 2012; Anaya-Gutiérrez et al. 2022); this confusion is not trivial, since secretory canals and cavities have been shown to have similar structural characteristics. Developmental studies have provided important information to understand the differences between similar secretory structures (Ramírez-Díaz et al. 2019), and their evolutionary, ecological, and systematic implications have been recognized (Orton 1955; Wake 1989; Ochoa-López et al. 2020). Therefore, this study aims to characterize and identify differences in the development patterns of secretory canals and cavities in Asteraceae, and thus provide more information to be able to differentiate them.

## Material and methods

### Taxon sampling

Two Asteraceae species growing naturally in the Pedregal de San Ángel Ecological Reserve (REPSA-UNAM) were selected, which previously identified the presence of canals or secretory cavities. *Tagetes tenuifolia* Cav. (Tageteae) and *Bidens odorata* Cav. (Coreopsideae) were chosen as study models, the first for having secretory cavities and the second for including canals in their mature leaves (Rivera et al. 2019; Martínez-Quezada et al. 2023). From each species, 10 individuals were selected, from which leaves in different stages of development were collected, from leaves located at the apex of the stem to fully expanded leaves. In the case of the smallest leaves (very close to the apex of the stem), they were removed together with the apical meristem, while in the case of expanded leaves, a lobe from the middle third of the blade was removed.

To compare the structural characteristics of fully developed secretory cavities within Tageteae, species from the genera *Adenophyllum*, *Bajacalia*, *Chrysactinia*, *Dyssodia*, *Haploesthes*, *Jaumea*, *Nicolletia*, *Pectis*, *Porophyllum*, another species of *Tagetes*, and *Thymohylla* were included in this study (Appendix 1). Samples of mature leaves without apparent damage from specimens deposited in the National Herbarium of Mexico (MEXU) and the University of Texas at Austin Herbarium (TEX) or collected in the field were selected.

### Microtechnique

The samples were fixed in glutaraldehyde for 24 h, after which time they were washed with distilled water and preserved in 30% ethanol. The fixed samples were dehydrated in a gradual series of ethanol until absolute ethanol, subsequently infiltrated, and included the tissues in glycol-methacrylate (Zarlavsky 2014). The included samples were mounted on wood sample holders and sectioned with a rotary microtome in the transverse and longitudinal plane at 3–5-μm thickness. The sections were stained with cresyl violet (Zarlavsky 2014) and mounted with synthetic resin; observations were made under the optical microscope.

Leaf samples of the rest of the species of Tageteae analyzed obtained from herbarium specimens, were pretreated according to Martínez-Quezada et al. (2023), while those from the field were fixed in formalin-acetic acid–ethanol (FAA; Ruzin 1999). All samples were rinsed and dehydrated in gradual ethanol series in a Leica automatic TP1020 changer and embedded in Paraplast (Leica). Transverse sections were sectioned with a rotary microtome (Leica RM2125RT) 10–16-μm thickness, stained with safranin-fast green and mounted with synthetic resin.

### Scanning electron microscopy (SEM)

Samples of mature lobes of *T. tenuifolia* and complete leaves of *Chrysactinia mexicana* were fixed in FAA (Ruzin 1999) and prepared for analysis with SEM observations. They were rinsed in distilled water and dehydrated in a gradual series of ethanol 50, 70, 96, and 100%, and then ultrasonified with chloroform for 15 min to remove the epicuticular waxes (Cota-Sánchez and Bomfim-Patrício 2010). Dehydrated samples were critical point dried with CO_2_, and mounted on aluminum specimen holders with double-sided tape to cover them with gold in a Hitachi-S-2460N sputter coater. Samples were observed under a Hitachi SU1510 (10 kV) at the Institute of Biology, UNAM.

## Results

**Fig. 1:**
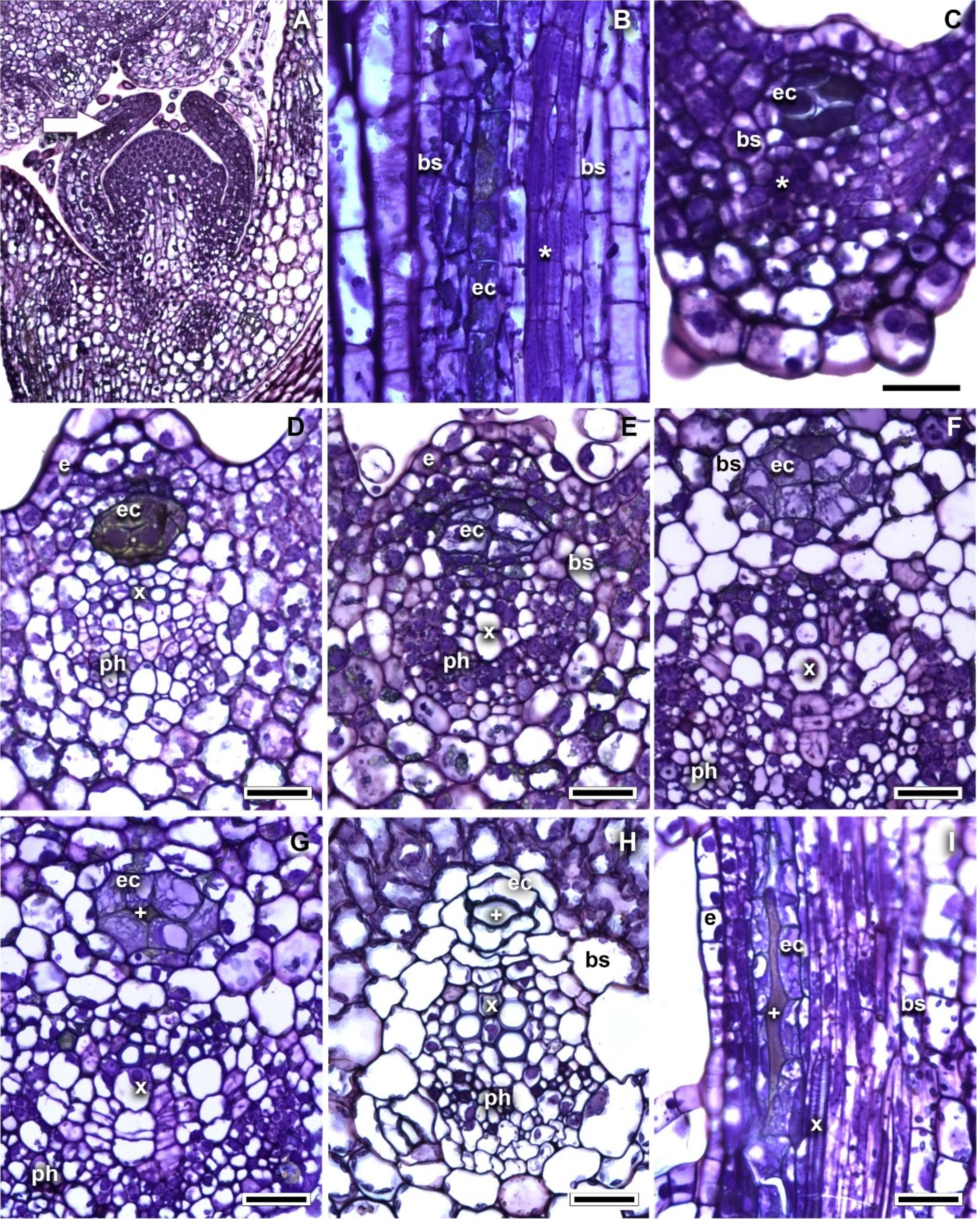
Canals development in *Bidens odorata*. **A** Stem apex in longitudinal section showing the first three pairs of leaf primordia and the first cells corresponding to the beginning of the secretory canals (arrow). **B**, **C** Differentiation of the procambial strand (*) and of the canal epithelial cells, longitudinal (ls) and transverse (ts) sections. **D** Final position of the epithelial cells with respect to the vascular bundle, locating between the bundle sheath and the xylem of the vascular bundle, ts. **E** Differentiation of canal sheath cells; epithelial cells are greater in size and highly vacuolated than the rest of the adjacent cells, ts. **F** The cells of the canal sheath start to grow and become clearer, which indicates a decrease in cellular metabolism, ts. **G**–**H** Development of the intercellular space that leads to the development of the canal lumen ( +), ts. **I** Canal lumen with content ( +), ls. Scale bar is 100 µm in A, 25 µm in **B**–**H**, 50 µm in **I**. bs, bundle sheath; e, epidermis; ec, epithelial cell; ph, phloem; x, xylem

The studied secretory structures appear in the early stages of leaf development, approximately between the second and third pair of developing leaves (Fig. 1A). When analyzing the leaf primordia of *Bidens odorata*, it was observed that the development of canals is closely associated with the differentiation of the vascular system. Once the procambial strand was differentiated, the epithelial cells were distinguished because they were larger and with a dense protoplast (Fig. 1B, C). The final position of the epithelial cells concerning the vascular bundle became evident once the first tracheary elements appeared, locating between the vascular bundle sheath and the xylem pole of the vascular bundle (Fig. 1D). At this time, differentiation of canal sheath cells began, epithelial cells continued to increase in size and became highly vacuolated compared with the adjacent cells, while cells of the canal sheath exhibit large nuclei (Fig. 1E). The cells of the canal sheath started to grow, and their cytoplasm was less dense indicating a decrease in cellular metabolism (Fig. 1F). In this development stage, in the central region of the cluster of epithelial cells, the middle lamella started to dissolve, giving rise to an intercellular space that leads to the formation of the canal lumen that fills with contents of the secondary metabolism (Fig. 1G). Due to the association of the canals with the vascular system, when the canals have completed their development, it is difficult to identify the cells of the bundle sheath. No modifications were observed in epidermal or mesophyll cells (Fig. 1H, I). It is important to mention that after the vascular system in the midvein is almost complete, one or two canals start to differentiate from the mesophyll (not shown).

**Fig. 2:**
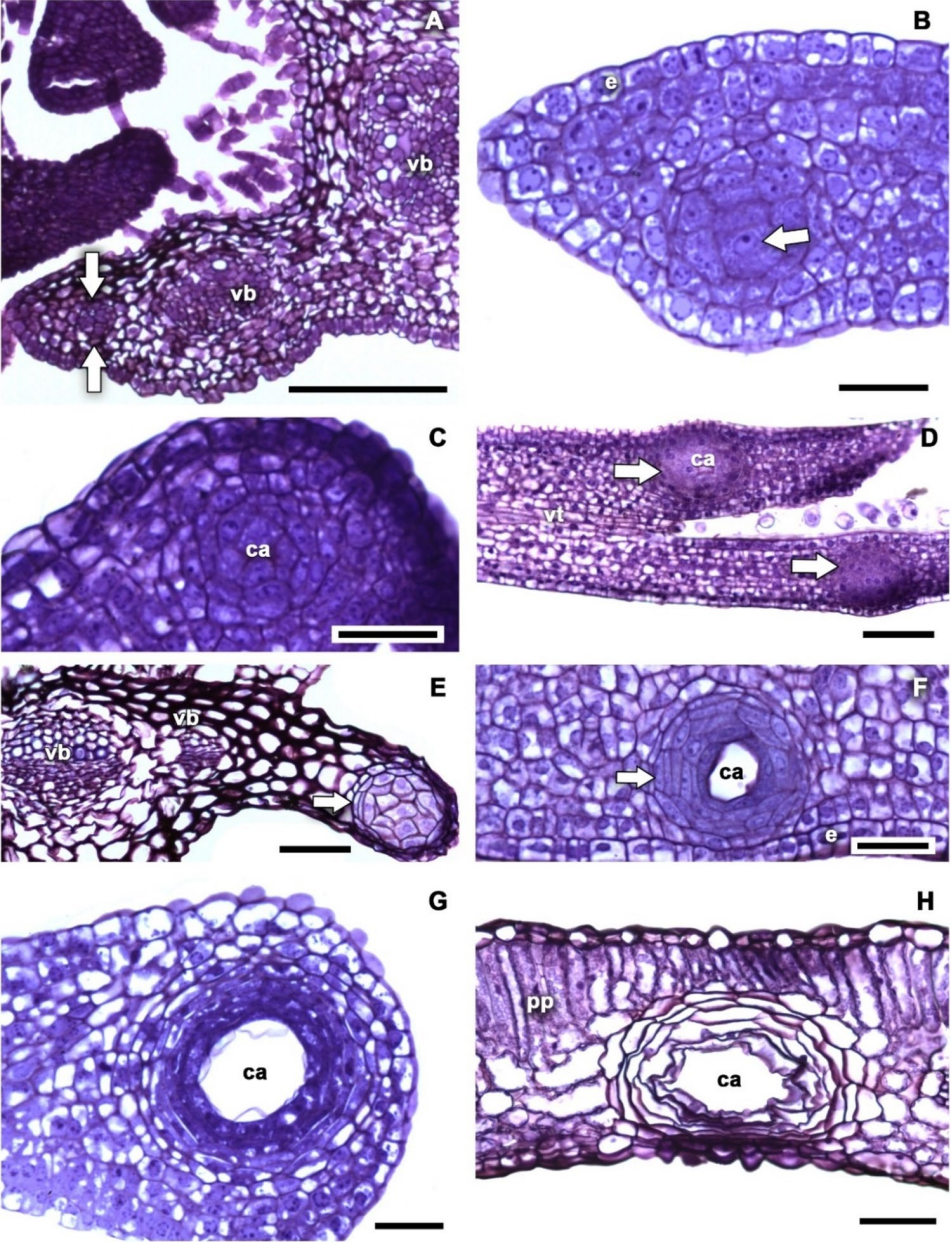
Secretory cavity development of *Tagetes tenuifolia* in transverse (ts) and longitudinal sections (ls). **A** Initiation of secretory cavity development as a cluster of cells with divisions in all planes (arrows), tr. **B** End of cell divisions in the periphery of the cavity while the central cells continue dividing without a defined pattern (arrow), ts. **C** Development of the intercellular space that leads to cavity lumen formation ( +), ts. **D** Development of two cavities in different lamina lobules once the upper cavity lumen started ( +), ls. **E** First periclinal divisions of the sheath cavity cells (arrow), ts. **F** Increased number of sheath cavity cells (arrow) and larger cavity lumen, ls. **G** Secretory epithelium cells become increasingly compact as the canal lumen grows, ts. **H** The ontogeny finishes when the multistratified epithelial cells, sheath cells, and cavity lumen are fully developed, ts. Scale bar is 100 µm in A, 25 µm in **B**, **C**, **F**, **G**, 50 µm in **D**, **E**, **H**. ca, cavity lumen; e, epidermis; pp, palisade parenchyma; vb, vascular bundle; vt, vascular tissue

**Fig. 3:**
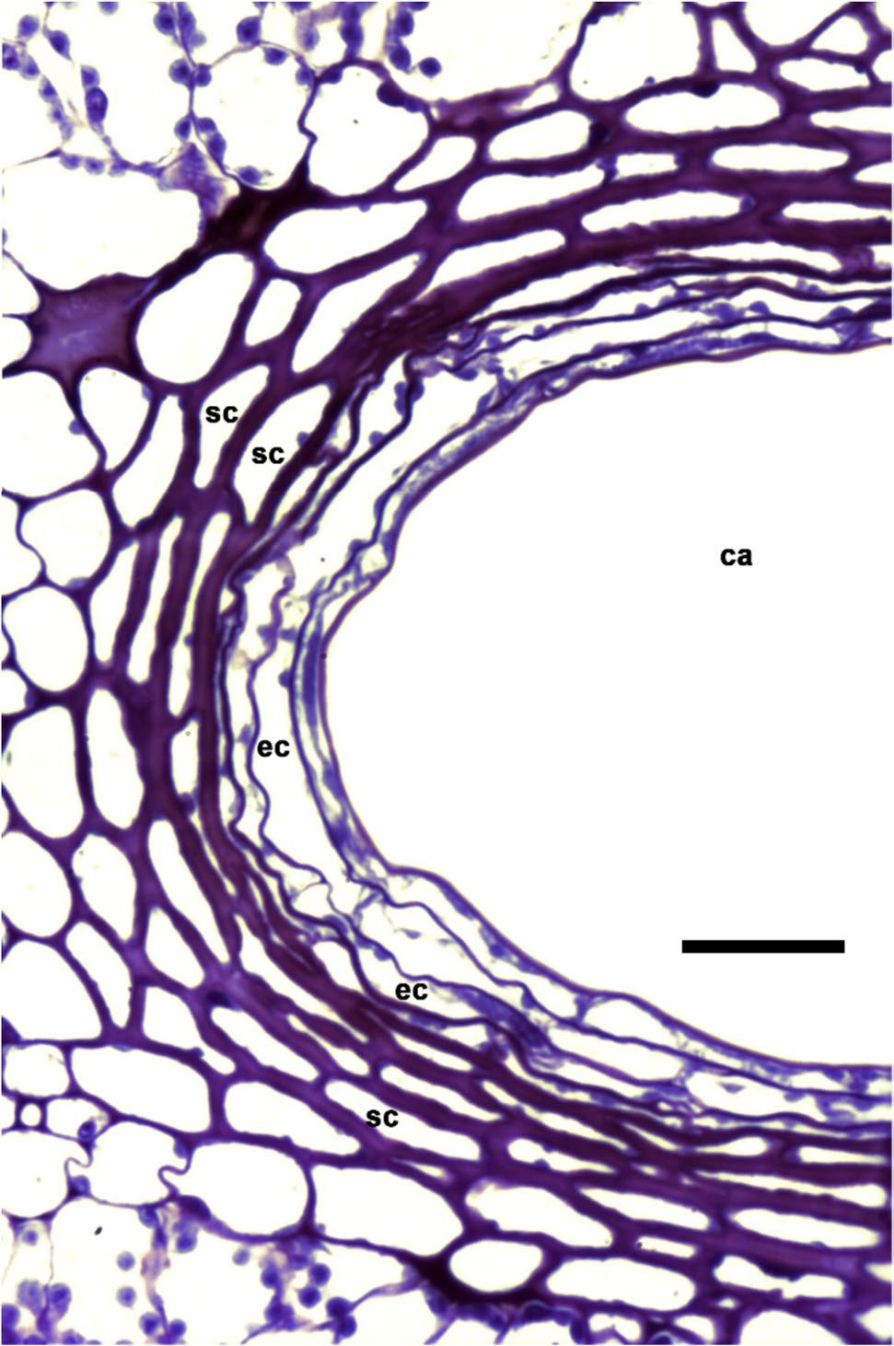
Structural characteristics in the fully developed secretory cavities of *Porophyllum ruderale*. Scale bar is 25 µm. ca, cavity lumen; ec, epithelial cell; sc, sheath cell

The pinnately lobed leaf primordia of *Tagetes tenuifolia* gradually elongates, and a secretory cavity develops towards the apex of each lobe. However, in the mesophyll, the secretory cavities begin to develop when the vascular bundles have almost completed their development. The secretory cavity initiates as a cluster of cells with divisions in all planes, forming a more or less spherical mass of cells (Fig. 2A–D). The cells on the periphery of the cluster seem to cease their cell divisions while the central cells continue dividing without a defined pattern. The new cells are larger than those that gave rise to them and have large nuclei (Fig. 2B, C). Once this occurred, the middle lamella dissolves to form an intercellular space, which gives rise to the lumen of the secretory cavity (Fig. 2C). In addition, more than three periclinal divisions are observed in the outer cells of the cavity, which make up the sheath of the cavity (Fig. 2E, F). As the lumen increases in size, the epithelial cells surrounding the cavity lumen become increasingly compact (Fig. 2G), while the cells at the periphery thicken their walls and the sheath of the cavity becomes conspicuous (Fig. 2H). The ontogeny of secretory cavities finishes when the multistratified epithelium and the cavity sheath are fully developed (Fig. 3). Epithelial cells in secretory cavities are functional, metabolically active, and have very thin cell walls, so, commonly, the inner strata of epithelial cells may detach due to an artifact of microtechnique.

**Fig. 4:**
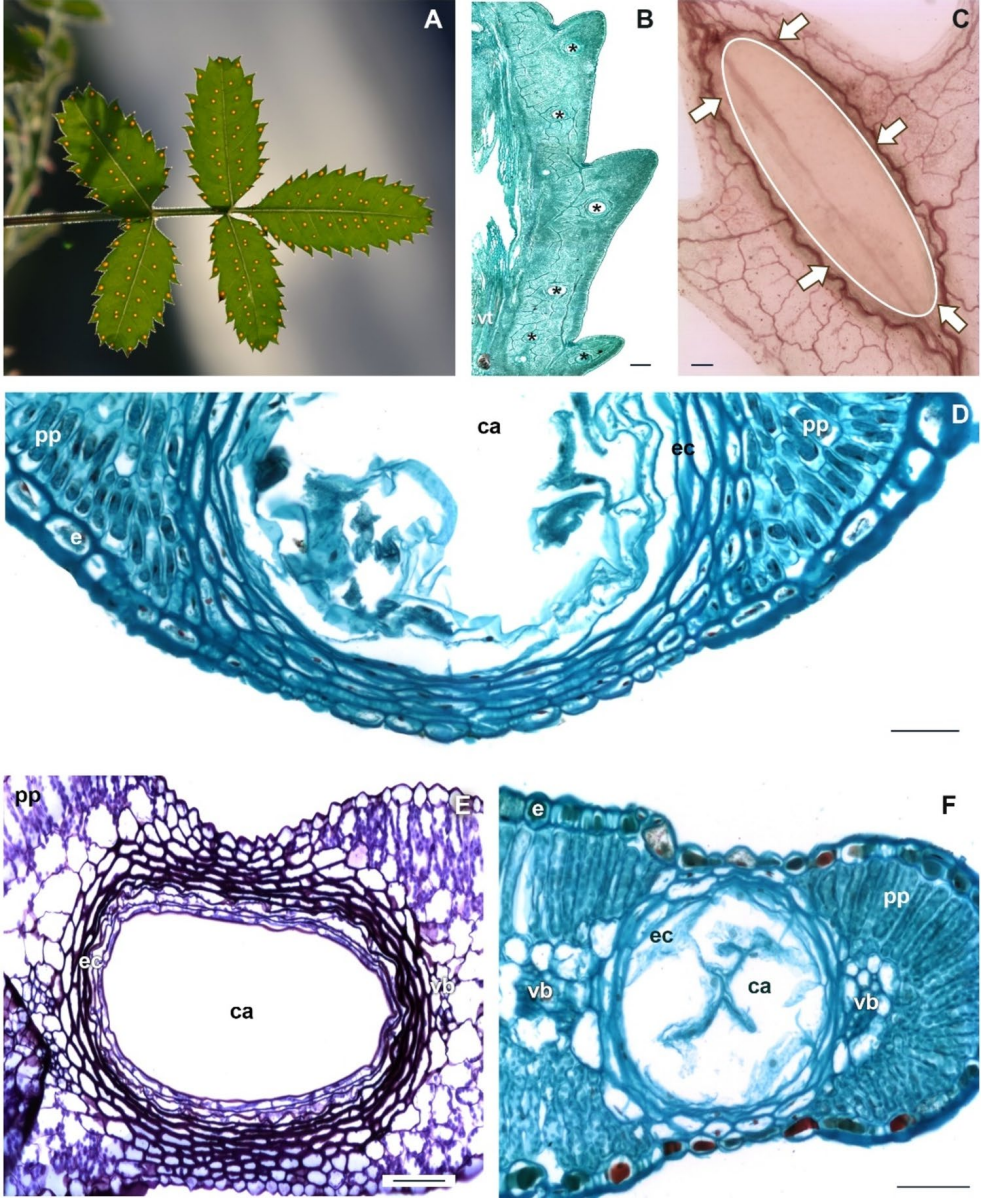
Structural modifications associated with the secretory cavities in some genera of Tageteae. **A** Secretory cavities in *Tagetes parryi*. **B** *T. tenuifolia*, paradermal section showing the secretory cavities in the blade. **C** *Adenophyllum cancellatum*, cleared leaf showing the secretory cavity delimited by the brownish oval and epithelial and sheath cells (arrows). **D** *Chrysactinia mexicana*, modifications in mesophyll and epidermis cells associated with the secretory cavity, ts. **E** *Porophyllum ruderale*, depression in the epidermal surface associated with the secretory cavity, ts. **F** *Dyssodia papposa*, depression in both epidermal surfaces associated with the secretory cavity and modifications in mesophyll, ts. Scale bar is 300 µm in B, 100 µm in C, 50 µm in **D**–**F**. ca, cavity lumen; e, epidermis; ec, epithelial cell, pp, palisade parenchyma; vb, vascular bundle, vt, vascular tissue

**Fig. 5:**
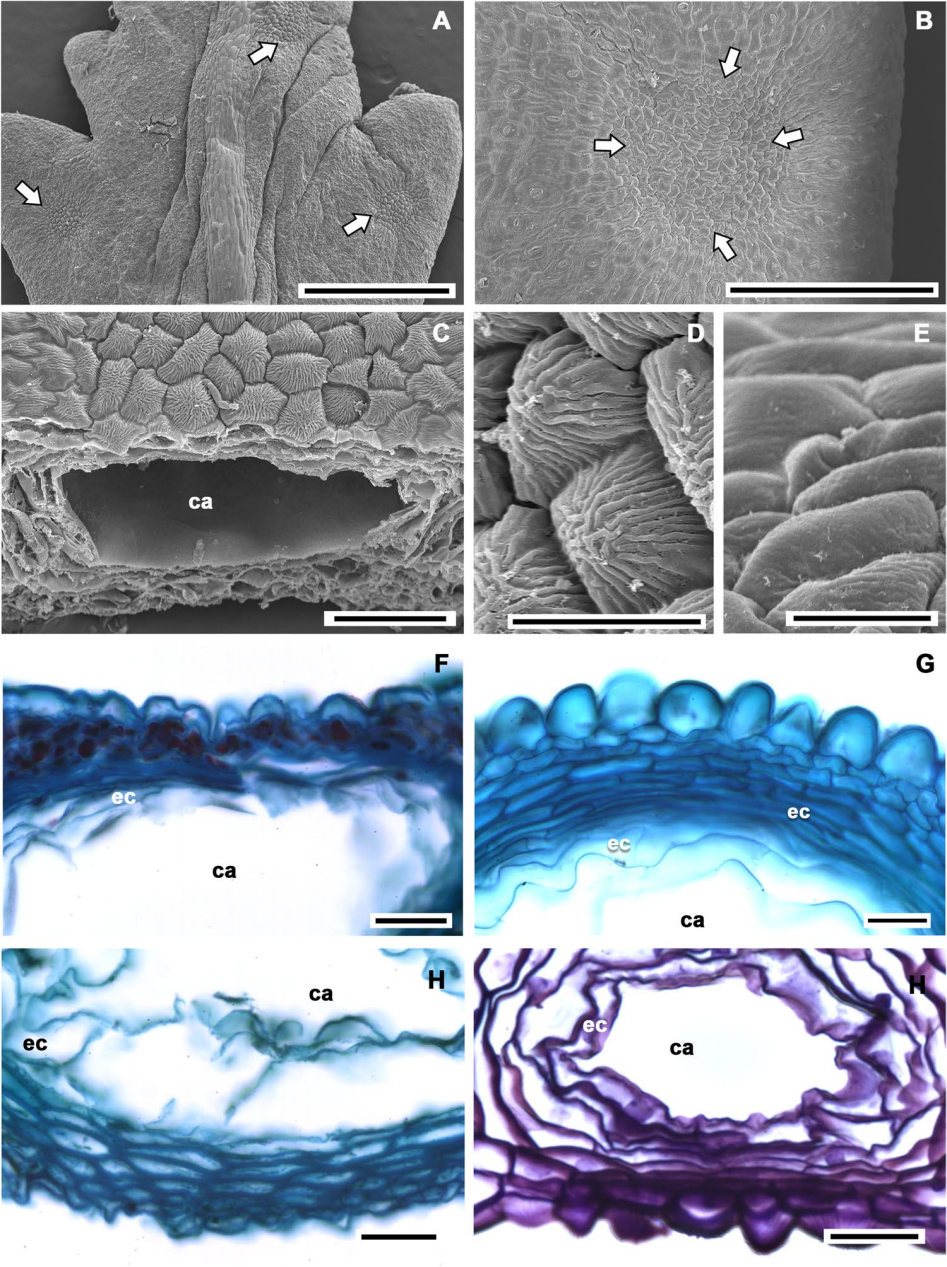
Structural modifications of the epidermal surface and transverse sections associated with the secretory cavities in some genera of Tageteae view with MEB and light microscopy. **A** *Tagetes tenuifolia*, adaxial surface, arrows point out the position of the secretory cavities. **B** *Chrysactinia mexicana*, abaxial surface, detail of secretory cavity position (arrows) showing the depression. **C** *T. tenuifolia*, detail of secretory cavity and associate adaxial epidermis. **D** *T. tenuifolia*, striate microrelief of epidermal cells associated with the secretory cavity. **E** *C. mexicana*, smooth microrelief of epidermal cells associated with the secretory cavity. **F** *T. foetidissima*, strongly convex outer periclinal wall of epidermal cells. **G** *Adenophyllum cancellatum*, strongly convexed outer periclinal wall of epidermal cells. **H** *Porophyllum viridiflorum*, conical outer periclinal wall of epidermal cells. **I** *T. tenuifolia*, conical outer periclinal wall of epidermal cells. Scale bar is 500 µm in **A**; 300 µm in **B**; 50 µm in **C**; 20 µm in **D**, **E**; 25 µm in **F**–**I**. ca, cavity lumen; ec, epithelial cell

In this work, members of three of the four subtribes of Tageteae were analyzed (Appendix 1), of which only the species included in the subtribe Pectidinae presented secretory cavities (Fig. 4). The secretory cavities in Pectidinae were large (even seen with the naked eye, Fig. 4A), round to oval (Fig. 4B, C), and presented a well-differentiated sheath variable in the number of cells strata, always being more than three (Fig. 4D), and generally, this has thicker walls compared to epithelial. Cavities occupy in most cases a great proportion of the leaf mesophyll; vascular bundles are displaced, and the palisade and spongy parenchyma may be missing; thus, the cavity sheath cells are almost in contact with the adaxial and abaxial epidermis (Fig. 4D–F). When this occurred, the leaf epidermis showed modifications in the regions associated with the secretory cavities for the rest of the epidermal surface (Fig. 5). In these cases, it was common the occurrence of a depression in the epidermis (Figs. 4E, F and 5A, B). In this depression of the epidermis, cells were smaller with thin outer periclinal walls (Fig. 5F–I), generally convex to conical (Fig. 5D–G), often with different types of microreliefs (papillose, striated, or smooth) about other epidermal cells (Fig. 5C, D, H) in the abaxial epidermis or in both.

## Discussion

Although secretory systems (particularly secretory structures) are found in many species among vascular plants, currently, there is no general consensus to standardize their nomenclature. This inconsistency in the use of the terminology has led to assigning several names to the same secretory structure, which makes it difficult to have a more precise overview of their diversity, making the establishment of homology hypotheses more complicated.

Those secretory structures with similar structural characteristics, as the case of canals and cavities, are often confused due to technical or interpretive issues (Kalachanis and Psaras 2005; García-Sánchez et al. 2012; Anaya-Gutiérrez et al. 2022). This is mainly due to their structural similarities, such as the presence of a secretory epithelium delimited in many cases by one or more strata of non-secretory cells forming a sheath (Crang et al. 2018). Likewise, due to their size and position, they are often referred in literature to as glands or pellucid glands (Lizarraga et al. 2017; Aguilar-Rodríguez et al. 2022), making the use of terms to differentiate them even more confusing. Although studies describing the ontogeny of secretory structures have been important in their delimitation (Bezerra et al. 2018), the boundaries defining a secretory cavity remain unclear.

For a long time, the main criterion for distinguishing between internal secretory structures with epithelial cells was the ontogenetic origin of their intercellular space (lumen), which was described as schizogenous or lysigenous (Esau 1977; Fahn 1982). Under this premise, the lumen of the canals would develop from the separation of the cells by the dissolution of the middle lamella (schizogenous origin), while in the secretory cavities that lumen will be of lysigenous origin, that is, it will be formed due to programmed cell death (Turner et al. 1998).

Secretory cavities have been reported mainly in Rutaceae and Myrtaceae (Fahn 1979), families where the greatest number of studies on the development of this structure has been concentrated. In the genus *Citrus* (Rutaceae), cavities have been reported in both vegetative and reproductive organs and the development of the lumen of these cavities has been described mainly as lysigenous (Knight et al. 2001; Rafiei and Rajaei 2007), but to a lesser extent, they have also been described as schizogenous. However, some authors even consider that the boundary between schizogenous and lysigenous cavities in *Citrus* is not clear, so they decided to describe them as schizo-lysigenous (Fahn 1988). Due to these inconsistencies, Turner et al. (1998) using various fixation methods and different histology embedding media determined that the lysogenous origin of the secretory cavities in *Citrus* corresponded to an artifact of histological processing, actually being of schizogenous origin, as later confirmed by Bosabalidis (2010).

The secretory cavities of Myrtaceae are very similar at a macroscopic level to those of Rutaceae (Kalachanis and Psaras 2005), often being described as pellucid glands. The most studied genus of this family is *Eucalyptus* and unlike Rutaceae; there is sufficient evidence of the dissolution mechanisms of the middle lamella that give way to the formation of an intercellular space without cell lysis (Carr and Carr 1970). The lumen of the pellucid glands (oil glands) of *Eucalyptus* has a schizogenous origin (Carr and Carr 1970). In the two Asteraceae species analyzed here, both the lumen of the canals and cavities have schizogenous development, and there are no signs of programmed cell death or cell lysis that will lead to the lumen of the secretory structures. Instead, the intercellular space started through the separation of the epithelial cells, which was probably mediated by the dissolution of the middle lamella, consistent with previous reports for *Tagetes minuta* (Del Fueyo 1986) and *Verbesina macrophylla* (Bezerra et al. 2018), but differing with *Porophyllum lanceolatum* described as lysigenous (Monteiro et al. 1995).

According to Knight et al. (2001), Bennici and Tani (2004), and Rafiei and Rajaei (2007), canals and secretory cavities originate from different promeristems. In Rutaceae, secretory cavities derive from meristematic cells of the protodermis and the fundamental meristem, while in Myrtaceae, they originate only from cells of the protodermis (Carr and Carr 1970; Kalachanis and Psaras 2005). In the case of *Bidens odorata*, the initiation of the epithelial cells occurs with the differentiation of the procambial cells; this position is maintained during the development of the leaf blade and coincides with the path of the vascular bundles in the veins of the leaf. Therefore, it can be inferred that the identity of the procambial cells at this point of development is already determined. These procambial cells will give rise to the epithelial cells of the canal and at the same time to the vascular system (Bezerra et al. 2018). On the other hand, the initiation of the epithelial cells in *Tagetes tenuifolia* cavities is not spatially or ontogenetically associated with the procambial cells, but rather they originate from cells of the fundamental meristem in very localized regions of the lamina (Fig. 4B), as occur with other species of this genus (Del Fueyo 1986). Notably, the cavities in the lobes of the blade were initiated after the vascular bundles were differentiated.

Some authors consider that length is another important difference between canals and secretory cavities, i.e., Ferreira Fernandes et al. (2018). Under the classic definition of canals, these would correspond to structures that form an elongated intercellular space, while cavities would correspond to more or less spherical and elliptical intercellular spaces, as reported for different regions of the plant body as the leaves or bark (Fahn 1979; Angyalossy et al. 2016). However, canals of different sizes have been observed in several species of some tribes of Asteraceae as in Astereae, Coreopsidae, Eupatorieae, and Tageteae; some are more or less elliptical and less than 30 μm and other elongated canals of more than 100 μm (Del Fueyo 1986; Martínez-Quezada et al. 2023); so, this variation in length is not a character that allows us to differentiate canals from cavities in leaves, at least in Asteraceae.

**Fig. 6:**
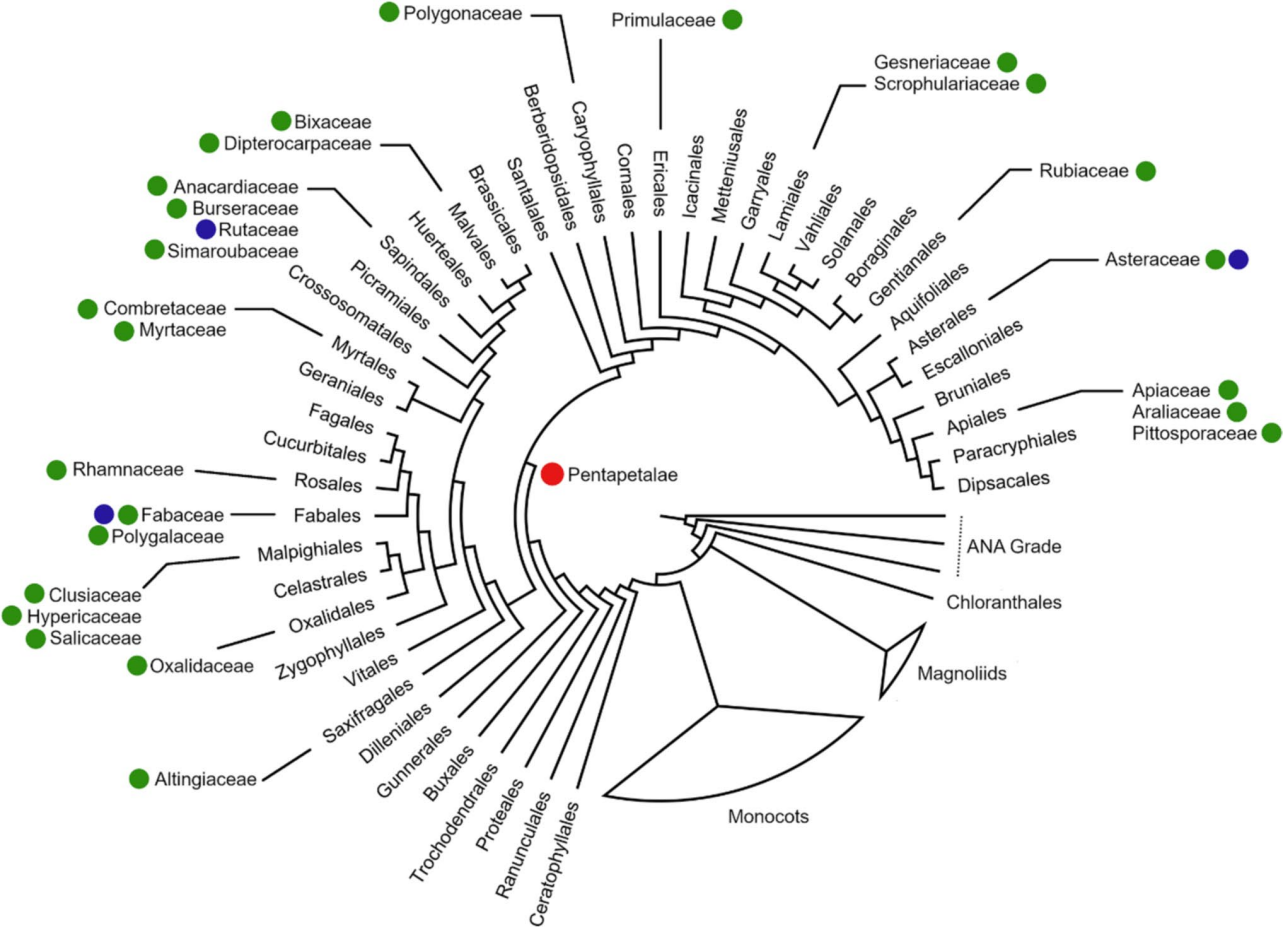
Phylogeny of angiosperms (APG IV) indicating the occurrence of canals and cavities in the Eudicots, following Metcalfe and Chalk (1950) and other authors. Red circles indicate the node position of Pentapetalae clade; green circles, canals; blue circles, cavities

Previously, Martínez-Quezada et al. (2023) highlighted that in Asteraceae, the number of strata of epithelial cells and the sheath are consistent differences that allow them to be delimited in the family (unistratified epithelium and sheath in the canals vs. multistratified epithelium and sheath in the case of cavities). Under this definition for Asteraceae, the pellucid glands or oil glands reported in Rutaceae effectively correspond to secretory cavities, while those reported for Myrtaceae correspond to short canals. One of the most comprehensive works about the vegetative anatomy of angiosperms is that of Metcalfe and Chalk (1950); according to these authors, secretory cavities are present in at least 36 families from 14 orders of Eudicots. However, using the structural characteristics of epithelial cells and sheath as a criterion to identify secretory cavities; this secretory structure is present only in members of Asteraceae, Fabaceae, and Rutaceae (Fig. 6).

Secretory cavities are large containers of products of secondary metabolism, which in most cases occupy the whole space of mesophyll between both adaxial and abaxial epidermis. In this study, we explore the structural modifications of epidermis that are present when secretory cavities are closely associated with the epidermal cells. These cells are smaller than in other regions of the epidermal surface and, in many of the analyzed species, are located on a depression of the epidermis, corresponding to the position of the secretory cavity. Epidermal cells associated with the secretory cavity showed differences mainly in the outer periclinal wall, which was generally thin and could be markedly convex to conical. Moreover, these epidermal cells associated with the cavity commonly varied in the microrelief compared to those not associated with the secretory cavity. These traits are present in other secretory structures as osmophores (Cabral de Melo et al. 2010; Fiorilo Possobom et al. 2015; Demarco 2017; Tölke et al. 2018; Francisco and Ascensão, 2024) that are implicated in the liberation of highly volatile compounds, like fragrances and perfumes. Epidermal modifications from certain areas of the leaf blade also occur in members of Fabaceae with secretory cavities and Rutaceae (Reis Mendes et al. 2019; Fortuna-Perez et al. 2021; Martínez-Quezada pers. obs.). So, they could be additional useful characteristics to differentiate cavities from canals, since in the latter, no modifications of the epidermis had been observed, a feature also mentioned by Simon et al. (2002) for *Tagetes minuta*. In members of the subtribe Pectidinae of Asteraceae, there is no evidence that substances were eliminated through epidermal cells. However, the production of volatile oils has been identified in different species of *Tagetes* (Russin et al. 1988; Díaz-Cedillo and Serrato-Cruz 2011); thus, further studies are needed to confirm that volatile oils are produced in the secretory cavities of other members of subtribe Pectidinae and that they are liberated through the epidermal cells.

Based on Metcalfe and Chalk (1950), it is clear that secretory canals are well represented in at least 18 eudicot families; however, many of the secretory canals reported in these families actually correspond to canals of different sizes and even to other secretory systems, such as secretory idioblasts, commonly mucilage cells. Applying the new reference framework to recognize canals and cavities proposed in this work, secretory canals are present in 23 families, all included in the Pentapetalae clade (Fig. 6; Appendix 2). The representativeness of canals in other lineages of flowering plants beyond the Eudicots has not been explored, but according to Prado and Demarco (2018), secretory canals are present in some monocot’s orders as Alismatales, Arecales, and Asparagales, so monocotyledonean lineages should be further explored in future studies.

Secretory cavities have evolved in lineages of angiosperms with a large representation of canals within vascular tissue (Vuillemin 1884; Van Tieghem 1885; Simon et al. 2002; Tölke et al. 2022) or extravascular (Carr and Carr 1970; Kalachanis and Psaras 2005). This also includes the simultaneous presence of both canals and cavities in the same organ (Del Fueyo 1986). For example, in the order Sapindales, which includes Rutaceae, canals are present in Anacardiaceae, Burseraceae, and Simaroubaceae (Tölke et al. 2022). In the same way, in the order Fabales, canals are predominant, particularly in members of the subfamily Faboideae of Fabaceae. In Asteraceae, the canals are present in at least 17 tribes, whereas secretory cavities are limited to Tageteae (Martínez-Quezada et al. 2023), so it can be determined that the canals are plesiomorphic concerning the secretory cavities, which only occurred in the subtribe Pectidinae of Tageteae (Martínez-Quezada et al. 2023). Because of this, we propose that secretory cavities could have arisen as a modification of extravascular canals in the evolutionary history of angiosperms, growing significantly in size relative to the thickness of the leaf. The occurrence of a multistratified thick-walled sheath probably provides structural support, perhaps to withstand greater pressure resulting from the accumulation of larger amounts of metabolites.

## Conclusions

Canals and secretory cavities in Asteraceae have a schizogenous origin. The development patterns of these types of secretory structures agree with those of groups such as Myrtaceae and Rutaceae. These two secretory structures in Asteraceae can develop from different promeristems, canals mostly from cells of the procambium, while the secretory cavities from the ground meristem. The most consistent structural characteristics that allow recognizing the canals from secretory cavities in Asteraceae correspond to the number of epithelial cell strata and the cavity sheath as reported in previous works, as well as differences in the epidermal cells associated to the cavity.

## Appendix 1

List of species of the tribe Tageteae presented in alphabetical order following this format: Subtribe: Genus and species: accession number of the herbarium specimens (Herbarium acronym: Herbario Nacional de México, MEXU, University of Texas at Austin TEX). BLT, B. Turner; DA, D. Álvarez; EM, E. Martínez; JGG, Jorge Gutiérrez Gallegos; JLP, José Luis Panero; JLV, José Luis Villaseñor; MS/MSM, Mario Suárez Mota; OHE, Oscar Hinojosa-Espinoza; RRM, Rosario Redonda-Martínez; YJCR, Yoceline J. Cadena Rodríguez

Flaveriinae: *Haploesthes greggii* A.Gray: *BLT*23-109 (TEX), *BLT*25-144(TEX). Jaumeinae: *Jaumea carnosa* (Less.) A.Gray: *JLP*8617 (TEX). Pectidinae: *Adenophyllum cancellatum* (Cass.) Villareal: *JLV*2218 (MEXU), *Adenophyllum glandulosum* (Cav.) Strother: *OHE630* (TEX), *Bajacalia crassifolia (S.Watson)* Loockerman, B.L.Turner & R.K.Jansen: *JLL*8239 (TEX), *Chrysactinia mexicana* A.Gray: *JGG*1622, *Dyssodia decipiens* (Bartl.)M.C.Johnst.: *MSM*147 (TEX), *Dyssodia papposa* (Vent). Hitchc.: *OHE*526 (MEXU), *Dyssodia pinnata* (Cav.) B.L.Rob.: *DA*15645 (MEXU), *JLV*2169 (MEXU), *Dyssodia tagetiflora* Lag.: *MSM*146 (TEX), *Nicolletia trifida* Rydb.: *JLP*2826 (TEX), *Pectis linifolia* L.: *MSM*372 (MEXU), *Pectis prostate* Cav.: *OHE*524 (MEXU), *Porophyllum coloratum* DC.: *JLV*2208 (MEXU), *Porophyllum macrocephalum* DC.: *FSL208* (TEX), *Porophyllum punctatum* S.F.Blake: *FSL*926 (TEX), *Porophyllum viridiflorum* DC.: YJCR97 (TEX), *Tagetes erecta* L.: MSM186 (TEX), *Tagetes filifolia* Lang.: DA14042 (MEXU), *EM*44693 (TEX), *Tagetes foetidissima* DC.: *JLV*2235 (MEXU), *Tagetes jaliscensis* Greenm.: EM44665 (TEX), *Tagetes lucida* Cav.: DA15646 (MEXU), JLV2175(MEXU), *Tagetes micrantha* Cav.: OHE434 (MEXU), RRM844 (MEXU), *Tagetes tenuifolia* Cav.: OHE474 (MEXU), *EM44662* (MEXU), *Thymophylla acerosa* (DC.) Strother: *JLV*2188 (MEXU), *Thymophylla aurantica* Rydb.: *DGM*619 (TEX).

## Appendix 2

Presence of canals and secretory cavities in Eudicot lineages, according to Metcalfe and Chalk (1950) and other authors

**Table:**

Order	Family	Canals	Secretory cavities	Reference
Apiales	Apiaceae	+	-	Metcalfe and Chalk 1950; Theobald (1967); Chuang (1970); Ghahreman and Amin (1996); Zarinkamar and Jalili (2004)
	Araliaceae	+	-	Metcalfe and Chalk (1950); De Villiers et al. 2010; Lotocka and Baczek (2024); DMMQ pers. obs. in *Schefflera*
	Pittosporaceae	+	-	Metcalfe and Chalk (1950); Boyko (2016); Myron et al. (2020)
Asterales	Asteraceae	+	+	Metcalfe and Chalk (1950); Del Fueyo (1986); Russin et al. (1988); Monteiro et al. (1995); García-Sánchez et al. (2012); Lizarraga et al. (2017); Naidoo et al. (2021); Aguilar-Rodríguez et al. (2022); Anaya-Gutiérrez et al. (2022); Martínez-Quezada et al. (2023)
Caryophyllales	Polygonaceae	+	-	Metcalfe and Chalk (1950); Lersten and Curtis (1992); Curtis and Lersten (1994)
Ericales	Primulaceae	+	-	Metcalfe and Chalk (1950); De Luna et al. (2013, 2014, 2017, 2018)
Fabales	Fabaceae	+	+	Metcalfe and Chalk (1950); Crow et al. (1997); Potgieter and Wessels (1998); Pinto et al. (2018); Reis Mendes et al. (2019); Fortuna-Perez et al. (2021)
	Polygalaceae	+	-	Metcalfe and Chalk (1950); Jorge et al. (2024)
Gentianales	Rubiaceae	+	-	Metcalfe and Chalk (1950); Cardoso Vieira et al. (2001)
Lamiales	Gesneriaceae	+	-	Metcalfe and Chalk (1950)
	Scrophulariaceae	+	-	Metcalfe and Chalk (1950); Karrfalt and Tomb (1983); Lersten and Beaman (1998); Lersten and Curtis 2001; Das et al. (2013)
Malpighiales	Clusiaceae	+	-	Metcalfe and Chalk (1950); Baroni Fornasiero et al. (2000); Perrone et al. (2013); Mattos Silva et al. (2019); Alencar et al. (2020)
	Hypericaceae	+	-	Metcalfe and Chalk (1950); Ciccarelli et al. (2001); Lotocka and Osinska (2010); Mejía-Agudelo et al. (2019)
	Salicaceae	+	-	Metcalfe and Chalk (1950); Thadeo et al. (2014)
Malvales	Bixaceae	+	-	Metcalfe and Chalk (1950); Freitas (2009)
	Dipterocarpaceae	+	-	Metcalfe and Chalk (1950); Srinual and Thammathaworn (2008); Noraini and Cutler (2009); Noraini et al. (2014)
Myrtales	Combretaceae	+	-	Metcalfe and Chalk (1950); Akinsulire et al. (2018); Waly et al. (2020)
	Myrtaceae	+	-	Metcalfe and Chalk (1950); Kalachanis and Psaras (2005); Retamales et al. (2014); Carneiro da Costa et al. (2020)
Oxalidales	Oxalidaceae	+	-	Metcalfe and Chalk (1950); Estelita-Teixeira (1982); Richetti et al. (2022)
Rosales	Rhamnaceae	+	-	Metcalfe and Chalk (1950); Colares and Arambarri (2008); Arsenijević et al. (2018); Iwamoto et al. (2023)
Sapindales	Anacardiaceae	+	-	Metcalfe and Chalk (1950); Royo et al. (2015); Tipmontiane et al. (2018); Tölke et al. (2021); Tölke et al. (2022); Mercado et al. (2024)
	Burseraceae	+	-	Metcalfe and Chalk (1950); Castro and Granada, (2010); Rosalem et al. (2017); Tölke et al. (2022)
	Rutaceae	-	+	Metcalfe and Chalk (1950); Liang et al. (2006); Zhou et al. (2014); Barbosa de Andrade et al. (2020); Shunmugam et al. (2021); Tölke et al. (2022)
	Simaroubaceae	+	-	Metcalfe and Chalk (1950); Saraiva et al. (2003); Tölke et al. (2022)
Saxifragales	Altingiaceae	+	-	Rodríguez-Sánchez et al. (2020)
